# Automated Multitier Tagging of Chinese Online Health Education Resources Using a Large Language Model: Development and Validation Study

**DOI:** 10.2196/83219

**Published:** 2025-12-17

**Authors:** Jialin Meng, Ruiming Dai, Xiaolan Huang, Yi Gu, Shixing Yan, Xiaoke Wang, Jingrong Gao, Tian-Tian Zhang

**Affiliations:** 1 School of Public Health Fudan University Shanghai China; 2 Shanghai Center for Emerging Technologies Governance in Medicine and Public Health Shanghai China; 3 Shanghai Municipal Center for Health Promotion Shanghai China

**Keywords:** health promotion, large language model, natural language processing, tagging, digital health, China, named entity recognition

## Abstract

**Background:**

Precision health promotion, which aims to tailor health messages to individual needs, is hampered by the lack of structured metadata in vast digital health resource libraries. This bottleneck prevents scalable, personalized content delivery and exacerbates information overload for the public.

**Objective:**

This study aimed to develop, deploy, and validate an automated tagging system using a large language model (LLM) to create the foundational metadata infrastructure required for tailored health communication at scale.

**Methods:**

We developed a comprehensive, 3-tier health promotion taxonomy (10 primary, 34 secondary, and 90,562 tertiary tags) using a hybrid Delphi and corpus-mining methodology. We then constructed a hybrid inference pipeline by fine-tuning a Baichuan2-7B LLM with low-rank adaptation for initial tag generation. This was then refined by a domain-specific named entity recognition model and standardized against a vector database. The system’s performance was evaluated against manual annotations from nonexpert staff on a test set of 1000 resources. We used a “no gold standard” framework, comparing the artificial intelligence–human (A-H) interrater reliability (IRR) with a supplemental human-human (H-H) IRR baseline and expert adjudication for cases where artificial intelligence provided additional tags (“AI Additive”).

**Results:**

The A-H agreement was moderate (Cohen κ=0.54, 95% CI 0.53-0.56; Jaccard similarity coefficient=0.48, 95% CI 0.46-0.50). Critically, this was higher than the baseline nonexpert H-H agreement (Cohen κ=0.32, 95% CI 0.29-0.35; Jaccard similarity coefficient=0.35, 95% CI 0.27-0.43). A granular analysis of disagreements revealed that in 15.9% (159/1000) of the cases, the “AI Additive” tags were not identified by human annotators. Expert adjudication of these cases confirmed that the “AI Additive” tags were correct and relevant with a precision of 90% (45/50; 95% CI 78.2%-96.7%).

**Conclusions:**

A fine-tuned LLM, integrated into a hybrid pipeline, can function as a powerful augmentation tool for health content annotation. The system’s consistency (A-H κ=0.54) was found to be superior to the baseline human workflow (H-H κ=0.32). By moving beyond simple automation to reliably identify relevant health topics missed by manual annotators with high, expert-validated accuracy, this study provides a robust technical and methodological blueprint for implementing artificial intelligence to enhance precision health communication in public health settings.

## Introduction

### Background

Effectively managing and disseminating the vast and ever-growing volume of digital health information is a fundamental challenge for modern public health systems worldwide. At the core of this challenge lies the problem of unstructured data, which hinders the ability to connect the right information to the right person at the right time. Health education resources (HERs) are defined in this study as the diverse set of digital assets consciously constructed to improve health literacy [1], which includes a wide array of formats such as text articles, short-form videos, audio clips, and slide decks [2]. Large HER repositories often lack the standardized metadata and consistent content labeling necessary for effective organization and dissemination [3]. This gap undermines the efficient retrieval, recommendation, and reuse of valuable content, exacerbating the problem of information overload for both clinicians and the public [4].

This challenge is particularly acute in the pursuit of precision health promotion, a paradigm that seeks to tailor health information according to an individual’s specific risk profile, behavior, and preferences [5]. Achieving this level of personalization at scale is contingent on a foundational layer of high-quality, finely structured content metadata. However, most health promotion platforms still rely on manual or simple rule-based tagging methods, which are labor-intensive, inconsistent, and difficult to scale [6], particularly across multimedia formats, such as text, video, and audio [7].

Although traditional machine learning pipelines have been applied to text classification, they often struggle to handle the implicit semantics and domain-specific nuances present in large heterogeneous HERs [8]. Recent advances in large language models (LLMs) have demonstrated powerful capabilities in abstractive summarization, entity recognition, and domain adaptation, thereby offering promising solutions to this structured content labeling bottleneck [9]. When coupled with expert-defined domain ontologies or taxonomies, LLMs have the potential to automate the complex task of assigning relevant and standardized tags to content. However, few empirical studies have validated the performance and real-world usability of such systems, particularly in the context of non-English (eg, Chinese language) HERs and deployment within public sector health agencies [10-12].

To address this gap, this study developed, implemented, and evaluated an artificial intelligence (AI)–powered, multitier tagging system for a large corpus of Chinese-language HERs. The aim was to enable high coverage, high consistency, and real-time tag assignment across multiple content modalities, thereby laying the technical foundation for a scalable, precision-driven health education delivery system.

### Objectives

This study aimed to develop a multitier tagging system using a fine-tuned LLM based on HERs from the Shanghai Municipal Center for Health Promotion. The objectives were as follows: (1) to design a 3-level tagging system for Chinese HERs, covering multiple resource modalities by leveraging their textual metadata; (2) to build and fine-tune an LLM-based pipeline to automate the assignment of these tags, thereby reducing the time and cognitive load required for manual annotation, improving tagging consistency across different content modalities, and achieving a target automation rate of at least 90%; and (3) to conduct a preliminary assessment of the system’s potential to improve retrieval efficiency and information equity by evaluating the quality, comprehensiveness, and expert-validated relevance of its automated tags.

### Related Work

#### Tailored Health Communication Frameworks

Tailored health communication refers to the strategic customization of health messages based on an individual’s characteristics, behaviors, and needs. This approach is known to enhance message salience, engagement, and behavioral outcomes [13]. Foundational theories such as the elaboration likelihood model and the health belief model have demonstrated the importance of aligning content with users’ cognitive and motivational profiles [14]. Systematic reviews have demonstrated that computer-tailored health communication can effectively increase physical activity, improve medication adherence, and empower patients to manage various chronic conditions [15]. However, although these theories support personalization at the message level, their practical application on digital platforms increasingly relies on structured metadata frameworks that enable real-time automated content delivery at scale [2].

#### Existing Mobile Health Tagging Efforts

Previous tagging efforts in mobile- and web-based health interventions have relied heavily on manually curated taxonomies or rule-based keyword systems. Similarly, Zhang et al [10] applied an ontology-driven annotation model to label health educational materials but noted the difficulty of maintaining semantic consistency across heterogeneous modalities. Although these approaches improve the metadata structure, they typically require substantial manual effort and lack generalizability, particularly in Chinese-language health communication contexts [16].

#### Limitations of Rule-Based and Shallow Machine Learning Approaches

Rule-based systems and conventional machine learning models (eg, support vector machines and decision trees) have been applied to the classification of HERs but exhibit several well-documented limitations, including limited adaptability, domain specificity, and poor performance on short or noisy text inputs [17]. These systems often depend on rigid feature engineering and fail to capture implicit contexts, a critical need in tagging multiformat, user-facing HERs. Conversely, LLMs have shown promise for abstractive summarization, named entity recognition (NER), and domain adaptation [18]. However, end-to-end validation of LLM-powered tagging pipelines, particularly within the public sector health infrastructure and multilingual multimedia settings, remains limited [17,19].

## Methods

### Taxonomy and Tagging System Design

We created a 3-level taxonomy for Chinese HERs by combining a top-down review of national public health standards with a bottom-up corpus-mining approach. The development process involved 2 main stages. An initial candidate pool of terms was generated by screening 22 official terminology sets and 15 peer-reviewed studies. The initial list was iteratively refined over the course of 2 Delphi rounds by a multidisciplinary expert panel (n=20; public health, health education, and informatics and AI, clinical specialties, and behavioral science). The panel’s task was to reach a consensus on the final hierarchical structure of the taxonomy. The degree of consensus was assessed using the scale-level Content Validity Index (S-CVI) and the Kendall W coefficient. A detailed description of the screening criteria, expert demographics, and item-level indices is provided in Multimedia Appendix 1.

### Data Collection

A dataset of 10,000 HERs was assembled in collaboration with the Shanghai Municipal Centre for Health Promotion (SMCHP). The corpus included a variety of modalities such as text articles, short-form videos, audio clips, and slide decks, each linked to human-curated key phrases provided by nonexpert staff. After preprocessing (deduplication, white space trimming, and removal of off-topic tags), the corpus was partitioned into training (n=7000), validation (n=2000), and testing (n=1000) sets.

The hold-out test set was further stratified by content modality to facilitate a nuanced performance evaluation. This resulted in 2 subsets: text-rich samples (n=689), comprising articles with abundant textual information, and text-sparse samples (n=311), consisting of multimedia resources with limited textual metadata, such as titles and brief descriptions.

### Model Development and Deployment

#### LLM Adaptation

The generative component was Baichuan2-7B, a 7-billion-parameter transformer model pretrained on a 2.6-trillion-token corpus. This model was selected for its state-of-the-art performance on Chinese language benchmarks and its open-source license, permitting research use. To facilitate efficient adaptation of the model to the health communication domain, we used low-rank adaptation (LoRA) [20], which is a parameter-efficient fine-tuning (PEFT) method. LoRA freezes the pretrained model weights and injects small, trainable, low-rank matrices into the transformer layers, reducing the number of trainable parameters to less than 0.2% and reducing memory use by approximately 40%. We configured the LoRA adaptation matrices with a rank (r) of 16 and a scaling factor (α) of 32. The training was performed on a single NVIDIA V100 GPU (80 GB) with mixed precision (fp16) for approximately 2 hours. We used the AdamW optimizer with an initial learning rate of 1 × 10^–4^, linear decay, and a batch size of 16.

#### Hybrid Inference Pipeline

**Figure 1:**
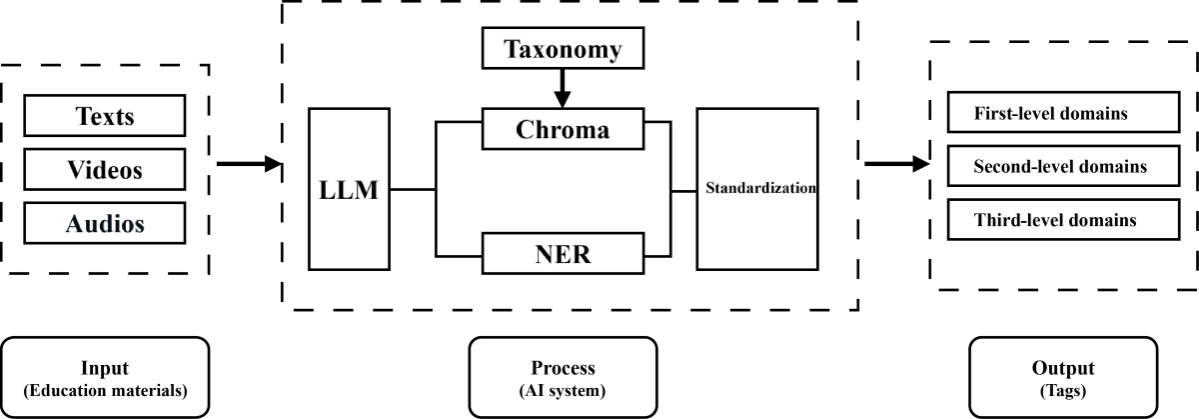
Workflow of the hybrid inference pipeline for automated tagging. AI: artificial intelligence; LLM: large language model; NER: named entity recognition.

At runtime, the system executes a 3-stage pipeline to ensure both thematic breadth and terminological precision: (1) the fine-tuned LLM summarizes the input text and proposes a set of candidate tags, (2) a domain-specific NER model filters these candidates to retain core thematic terms, and (3) the surviving terms are mapped to canonical labels in the taxonomy via a cosine similarity search within a Chroma DB vector store. This vector database was preloaded with embeddings for all concepts from our final taxonomy to ensure that all final outputs were standardized (see Figure 1).

#### System Deployment

The final model was packaged as a web service and deployed as a RESTful application programming interface (API) for integration into the live content management platform of the SMCHP, using Docker for containerization, Flask for API handling, and CUDA for graphics processing unit (GPU) acceleration. The model development process is detailed in Multimedia Appendix 2.

### Evaluation Framework and Baseline Creation

Our evaluation framework was designed to rigorously assess the AI system’s performance against a human baseline and to contextualize this performance against a nonexpert reliability benchmark. The evaluation involved a test set of 1000 documents and 3 distinct groups of raters.

#### Rater Population Definitions

The rater groups are defined as follows:

1. Nonexpert annotators: These were 2 original staff members (health management professionals without clinical expertise) who provided the initial free-text annotations. They were also rerecruited for the separate human-human (H-H) reliability study.
2. Expert tag designers: These were authors (JM and XH) who are experts in our taxonomy structure. Their role was to map the nonexpert free-text to the formal taxonomy.
3. AI system: This is the hybrid LLM-based inference pipeline.

#### Workflow 1: AI-Human Baseline Creation

To create the primary performance baseline (Human Label A), we followed a 4-step process:

1. Nonexpert free-text annotation: The 2 nonexpert annotators independently annotated the n=1000 test set documents, providing free-text keywords (phrases) based on their interpretation. They did not use the 90,567-term taxonomy.
2. Expert standardization: To create a valid, structured baseline, these free-text keywords were then manually mapped to the canonical 90,567-term taxonomy. This mapping was performed by the 2 independent expert tag designers (JM and XH), who were blinded to the AI system’s output. This rigorous process converted the unstructured keywords into the “Human-Standardized Labels” (Human Label A).
3. AI annotation: Concurrently, the AI system processed the original n=1000 documents (not the free-text) to produce the “AI-Generated Labels” (AI Label B).
4. Artificial intelligence–human (A-H) Comparison: The AI’s output (Label B) was then compared against the human-standardized baseline (Label A) to assess performance (see the Statistical Analysis subsection).

#### Workflow 2: Human-Human (H-H) Reliability Baseline

To address the editor’s requirement and provide an essential context for interpreting the A-H reliability, we conducted a formal human-human (H-H) interrater reliability (IRR) study:

1. Sampling: We drew a stratified random sample of n=100 documents from the n=1000 test set (text-rich, n=69, and text-sparse, n=31).
2. Procedure: We recontacted the 2 original nonexpert annotators (raters 1 and 2), who did not engage in the development of the taxonomy after their first round of annotation. Following training on the taxonomy interface and a 5-document pilot test, the 2 raters independently annotated the n=100 sample documents by selecting tags directly from the 90,567-term taxonomy. Raters were blind to each other’s annotations and all other study baselines. The outputs from this workflow were “Non-expert Label C” (from Rater 1) and “Non-expert Label D” (from Rater 2).

### Evaluation Metrics and Statistical Analysis

#### Evaluation Metrics

Our evaluation framework was designed for a real-world scenario lacking a “gold standard,” which centered on IRR and expert adjudication [21]. The evaluation methodology consisted of the following metrics:

1. IRR (A-H vs H-H): To measure the degree of consensus, we used Cohen κ and the Jaccard similarity coefficient. Cohen κ was used to measure the level of agreement beyond what would be expected by chance.
  A-H reliability (n=1000): We first measured the agreement between the AI system (Rater 1) and the legacy nonexpert human baseline (Rater 2) across the entire 1000-document test set.
  H-H reliability (n=100): To contextualize the A-H Cohen κ, we conducted a supplemental H-H IRR study. We randomly sampled 100 documents from the test set. A total of 2 original nonexpert staff annotators (Rater 1 and Rater 2), representative of the legacy workflow, independently tagged these 100 documents using the same guidelines. We then calculated the IRR between them.

To ensure a fair comparison, both A-H and H-H κ calculations were performed using the same methodology. We first identified the union of all unique labels produced by any rater (AI or human) across the 1000-document A-H test set. This resulted in a comparable set of 483 unique labels. The Cohen κ calculations were then performed by treating humans and AI as 2 raters across all possible document-label pairs (1000 documents × 483 labels for A-H and 100 documents × 483 labels for H-H) to construct the required contingency tables.

The Jaccard similarity coefficient, a complementary metric well-suited for multilabel tasks, was calculated to measure the overlap between the sets of labels assigned by the human and AI rater for each document. The final score was macroaveraged across 1000 documents in the test set.

1. Analysis of disagreement patterns: To understand the nature of the disagreements, we conducted a granular analysis by categorizing the annotation patterns of all 1000 documents into four distinct types: (1) complete agreement, (2) AI additive, (3) human additive, and (4) partial agreement or conflict.
2. Expert adjudication of AI-additive annotations: To assess the quality and validity of the additional information provided by the AI system, the “AI Additive” cases underwent expert adjudication. A random sample of 50 documents from this category was selected. For each case, a domain expert (senior author of this study) reviewed the source material and additional tags generated by the AI to determine whether they were correct and relevant, followed by the calculation of adjudicated precision.
3. Processing success rate: The proportion of resources for which the API successfully returned a nonempty set of tags within the operational timeout period. This metric is not a primary performance outcome and should not be interpreted as content-level correctness; our primary evaluation relies on IRR (A-H and H-H) and expert adjudication

#### Statistical Analysis

All statistical analyses were performed using Python (version 3.9; Python Software Foundation) and the *statsmodels* (version 0.13.2) and *scipy* (version 1.7.3) libraries. All key point estimates are reported with their 95% CIs. CIs for proportions, such as the adjudicated precision and the percentage of AI additive cases, were calculated using the Wilson score interval method, which is robust for proportions near 0 or 1. The CIs for macroaveraged metrics such as Jaccard similarity were calculated based on the SE of the mean. Furthermore, CIs for Cohen κ were computed using the asymptotic SE derived from the contingency table, as implemented in the statsmodels library. The statistical significance of differences in metrics between modalities (eg, text-rich vs text-sparse) was assessed by conducting a 2-sample *z* test for proportions or an independent samples *t* test for means, with a significance level set at *P*<.05.

### System Robustness and Feasibility

To validate real-world applicability, the final model was packaged as a web service and deployed as a RESTful API for integration into the SMCHP content management platform. The service architecture uses Docker for containerization, a Flask-based API for handling inference requests, and CUDA for GPU acceleration. The API workflow is structured as follows: (1) the frontend initiates a labeling request and sends it to the backend, (2) the backend invokes an automated tagging web service, (3) the tagging model returns standardized labels, and (4) the backend processes these labels and renders them on the frontend user interface (see Figure 1). This workflow enables the platform user interface (workers from the SMCHP) to send a resource to the backend, which invokes the tagging service and returns standardized labels for processing and display.

### Ethical Considerations

This study was based on a secondary analysis of a deidentified dataset of publicly available HERs provided by the SMCHP. The data contained neither any personal health information nor any other personally identifiable information. No compensation was provided to any individual for this secondary analysis. No images with identifiable individuals were included; therefore, no additional image-use consent procedures were required. According to institutional policies regarding the use of nonidentifiable public data for research purposes, this study was exempt from a formal institutional review board assessment. The IRB exemption policy referred to “National Health Commission Science and Education Development Document (2023) No. 4,” whose title is “Notice on Issuing the Ethical Review Measures for Life Sciences and Medical Research Involving Human Subjects” [22].

## Results

### Taxonomy Development and Validation

The expert-led development process generated a comprehensive 3-level hierarchical taxonomy of public health information. After the Delphi procedure, the final tag library comprised 10 first-level domains (L1), 34 second-level domains (L2), and 90,562 third-level concepts (L3). The vocabulary was derived from 10 national public health standards, a set of 20 official nomenclatures, and the SMCHP’s existing 3-level practice taxonomy, all of which were harmonized through expert Delphi rounds before being uploaded to the Chroma vector store (see Table 1; the examples of L3 labels illustrate the individual-level features for the health education audience based on specific conditions, demographics, or needs). Content validity was the scale-level Content Validity Index by the averaging method (S-CVI/Ave)=0.91; IRR reached Kendall W=0.78, meeting the prespecified stopping rule.

**Table 1:** Overview of the 3-tier health education taxonomy.

Primary category (L1)	Examples of secondary categories (L2)	Examples of tertiary tags (L3)
Disease prevention	Chronic noncommunicable disease prevention; communicable disease prevention; screening and early detection.	Prediabetes intervention; after coronary artery bypass graft surgery; human papillomavirus (HPV) vaccine;
Disease care and treatment	Symptoms and signs; diseases or conditions; medications; diagnostic and therapeutic procedures (tests and surgery)	Abnormal glucose tolerance test; coagulation abnormalities; confusion; impaired executive function; intravascular ultrasound
Rehabilitation and convalescence	Rehabilitation care; convalescent care; functional training	Orthopedic rehabilitation; cognitive rehabilitation; stroke rehabilitation
Healthy living	Nutrition and diet; smoking or alcohol cessation; physical activity; mental health	Visual impairment; personal hygiene; physical activity intensity; interpersonal conflict; mood disorder; child passenger safety
Health skills	First aid and emergency; health management; self-monitoring skills	Cardiopulmonary resuscitation (CPR); blood pressure monitoring; breast self-examination
Health policy and administration	Health policy and regulation; health education or promotion programs	Immunization program; outpatient reimbursement; cross‑region medical insurance reimbursement
Health culture	Folk health knowledge; traditional health practices	Tuina (message therapy); Chinese herbal paste
Population health	Population health	Resident health records; free medical consultation or clinic; community health services
Specific groups	Sex groups; age groups; occupational groups	Infants; adolescents; elderly; pregnant women; employed persons; retired
Professional groups	Public health; clinical medicine; nursing; traditional Chinese medicine	Radiology; inspection; Chinese acupuncture

### System Performance and Interrater Reliability

To assess the AI system performance against the nonexpert human annotations, we first established the H-H reliability baseline to quantify the typical consistency of the manual workflow. The analysis of 100 independently tagged documents by 2 nonexpert raters yielded a Cohen κ of 0.32 (95% CI 0.29-0.35) and a macroaveraged Jaccard similarity of 0.35 (95% CI 0.27-0.43). This indicates that the legacy human workflow has a “Fair” to “Moderate” and relatively unstable level of internal consistency.

Then, we compared the AI system’s output to the human annotation across the full 1000-document test set by treating them as 2 independent raters. This A-H analysis yielded an overall Cohen κ of 0.54 (95% CI 0.53-0.56) and a macroaveraged Jaccard similarity of 0.48 (95% CI 0.46-0.50). Critically, the A-H agreement (Cohen κ=0.54) was significantly higher than the H-H baseline agreement (Cohen κ=0.33). This finding suggests that the AI system not only learned the underlying logic of the manual workflow but also applied that logic with a higher degree of consistency than the nonexpert human annotators themselves. Table 2 shows that the agreement was substantially higher for text-rich articles than for text-sparse multimedia items.

To further understand the nature of these disagreements, we categorized the annotation patterns for all 1000 documents based on the relationship between the tag sets produced by the human annotator and the AI system. The distributions of these patterns are presented in Table 3.

**Table 2:** Overall performance metrics of the system on the test set.

Metric			H-H^a^ baseline (N=100)^b^		A-H^c^ system (N=1000)
**Overall**					
	Cohen κ (95% CI)	0.32 (0.29-0.35)		0.54 (0.53-0.56)	
	Jaccard similarity (macroaverage; 95% CI)	0.35 (0.27-0.43)		0.48 (0.46-0.50)	
**Text-rich samples (n=689 for A-H; n=69 for H-H)**					
	Cohen κ (95% CI)	0.38 (0.34-0.41)		0.62 (0.58-0.65)	
	Jaccard similarity (macroaverage; 95% CI)	0.39 (0.3-0.49)		0.56 (0.53-0.58)	
**Text-sparse samples (n=311 for A-H; n=31 for H-H)**					
	Cohen κ (95% CI)	0.23 (0.19-0.27)		0.44 (0.39-0.49)	
	Jaccard similarity (macroaverage; 95% CI)	0.27 (0.16-0.38)		0.39 (0.35-0.42)	

^a^H-H: human-human.

^b^H-H baseline metrics and CIs were calculated on the n=100 subset. Cohen κ CIs are based on the asymptotic SE from the n_docs * n_labels (483) contingency table. Jaccard CIs are based on the SE of the mean across documents.

^c^A-H: artificial intelligence–human.

**Table 3:** Patterns of agreement and disagreement across 1000 samples.

Annotation pattern	Sample count (N=1000), n (%)	Description
Complete agreement	583 (58.3)	The AI^a^ system and the human annotator produced identical sets of tags.
AI additive	159 (15.9)	The AI system included all human-assigned tags and added at least one new, relevant tag.
Human additive	85 (8.5)	The human annotator’s tag set was a superset of the AI’s tag set.
Partial agreement or conflict	173 (17.3)	The tag sets had some overlap but also contained unique or conflicting tags.

^a^AI: artificial intelligence.

### Qualitative Case Study and Human Comparison

The analysis of disagreement patterns (see Table 3) revealed that while “Partial agreement or conflict” was the most frequent type of disagreement (17.3%, 173/1000), the “AI Additive” pattern was the most critical for assessing the system’s value as an augmentation tool. This pattern, where the AI system provided more comprehensive thematic coverage than the human annotator, was also highly prevalent, occurring in 15.9% (159/1000, 95% CI 13.7%-18.3%) of all cases.

Therefore, our qualitative validation focused on this specific category to determine the reliability of the AI’s supplementary contributions. A random sample of 50 documents from the 159 “AI Additive” cases was selected for expert adjudication. For each case, a domain expert (senior author of this study) reviewed the source material, as well as additional tags, to determine their accuracy and relevance. The results were compelling: the expert review determined that in 90% (45/50; 95% CI 78.2%-96.7%) of the sampled cases, the additional tags provided by the AI system were both correct and relevant to the health topic. This high adjudicated precision strongly indicates that the AI system not only provides a broader thematic coverage but also has a high degree of accuracy. This finding reframes the system’s role from a simple automation tool to a powerful augmentation instrument capable of enhancing the depth and quality of health education content annotation, beyond the level achieved by nonexpert human staff.

### System Deployment and Operational Feasibility

The final model was successfully packaged as a web service and deployed as a RESTful API for integration into the live content management platform of the SMCHP. In the production environment, the system demonstrated high operational robustness. The system achieved an overall processing success rate of 94.8% (948/1000) on the test set. Performance varied by modality, with a rate of 97.4% (671/689) for text-rich samples and 89.1% (277/311) for text-sparse multimedia samples; this difference was significant (2-sample proportion *z* test; *P*<.001).

Furthermore, the system demonstrated high technical feasibility, achieving a median end-to-end processing latency of less than one second per resource. This performance met the operational requirements for real-time content ingestion and processing workflows, confirming the system’s readiness for practical large-scale applications in a public health setting.

## Discussion

### Principal Findings

This study successfully developed, validated, and deployed a hybrid AI pipeline for the automated, multilevel tagging of HERs within a live municipal public health platform. The core challenge of this study was methodological: how to rigorously evaluate an AI system in a real-world setting where no “gold standard” exists, and the human baseline is known to be imperfect.

To solve this, we adopted a robust evaluation framework centered on IRR. We first established an H-H baseline, which revealed a “Fair” to “Moderate” level of consistency (Cohen κ=0.32) among the nonexpert staff who represent the legacy manual workflow. We then compared the AI system’s output against this human baseline (A-H), which yielded a significantly higher “Moderate” agreement (κ=0.54). This is our first principal finding: the AI system not only had learned a significant portion of the logic of the human workflow but also applied it with statistically superior consistency than the humans themselves.

Our second principal finding was revealed through a granular analysis of these disagreements. Critically, in a substantial portion of the cases (159/1000, 15.9%), the AI system provided more comprehensive thematic coverage by identifying additional relevant topics that were missed by the human annotator (the “AI Additive” pattern). To validate the quality of these supplementary tags, expert adjudication was performed on a random sample of these cases. The results were compelling: the expert confirmed that the AI’s additional tags were correct and relevant in 90% (95% CI 78.2%-96.7%) of the cases. This high adjudicated precision provides strong evidence that the system functions not merely as an automation tool but also as a powerful augmentation instrument that can enhance the depth and quality of HERs annotations, surpassing the capabilities of nonexpert staff.

### Strengths and Innovations

The primary strength of this study is its end-to-end design, ranging from the rigorous expert-led development of a large-scale taxonomy to the successful deployment and methodologically sound evaluation of an AI system in a real-world operational environment. This study presents several key innovations. First, the hybrid technical architecture, which combines a parameter-efficient fine-tuned LLM with a vector database for standardization, was proven to be both effective and efficient for a large-scale ontology of over 90,000 terms. Second, our use of PEFT via LoRA demonstrated a computationally feasible approach for adapting a 7-billion–parameter model for a highly specific task, making the system maintainable for public health agencies with limited GPU capacity.

Third, and most significantly, this study contributes a robust evaluation framework for validating AI systems in real-world settings where a perfect “gold standard” is unavailable. By deliberately moving beyond simplistic or potentially misleading metrics, such as raw automation rates or accuracy against an imperfect baseline, and instead using IRR analysis coupled with expert adjudication of disagreements, we provide a more honest and insightful assessment of AI’s true value as an augmentation tool [23]. This methodological approach is critical for translating AI from theory to practice in complex, real-world domains.

### Implications for Health Promotion and Information Equity

The successful deployment and validation of this AI-powered tagging system have significant implications for health promotion, fundamentally shifting the paradigm from content delivery to knowledge engineering—that is, the systematic structuring of health information to make it computable and intelligently accessible. By deconstructing each unstructured HER as a set of discrete, interoperable units via granular L3 tags, our system transforms the content repository from a static collection of documents into a dynamic, queryable knowledge base. The power of this architecture is realized not through single tags but through their combinatorial application, which allows for the precise characterization and retrieval of content that aligns with the multifaceted profiles of specific audiences. For instance, consider the caregiver of an older adult’s stroke survivor. By querying for the intersection of tags, such as older adults (from “Specific groups”), stroke rehabilitation (from “Rehabilitation and convalescence”), and blood pressure monitoring (from “Health skills”), the system can dynamically assemble a holistic and contextually relevant package of resources—a task that would be difficult to achieve through traditional keyword searches alone. This combinatorial approach enables a truly person-centered model, wherein the system can infer and serve the user’s holistic informational needs.

Furthermore, this study provides a potential solution to the challenge of health information equity [24]. The digital divide concerns not only access to technology but also the ability to find relevant information within an overwhelming sea of content [25]. By creating a consistently and comprehensively tagged corpus, our system enhances the discovery of vital HERs for diverse populations. These structured data are also invaluable for public health surveillance and what the World Health Organization (WHO) terms “infodemic management” [26]. This aligns with international trends where AI-driven analysis of large-scale health data is increasingly used to inform policy. For example, the US Centers for Disease Control and Prevention uses its BioSense platform, which uses machine learning to analyze real-time, unstructured data to detect and monitor disease outbreaks, thereby enabling faster response and resource allocation [27]. Similarly, the WHO’s Early AI-supported Response with Social Listening (EARS) platform uses AI to analyze public narratives on social media, allowing health authorities to rapidly identify community health concerns, address information gaps, and counteract misinformation [28-30]. Our system provides the foundational data infrastructure for enabling similar data-driven public health governance.

Our findings also redefine AI’s role in public health workflow, not as a replacement for human expertise but as a collaborative partner, a concept the American Medical Association refers to as augmented intelligence [31]. Our system exemplifies this by automating the laborious task of initial tagging with high reliability, allowing human editors to focus on higher-level strategic tasks such as content validation, campaign design, and addressing complex health queries. This human-in-the-loop model is critical for building trust and ensuring the responsible deployment of AI in safety-critical domains such as public health.

However, the potential health literacy gap between the expert-derived logic embedded in our AI system and the comprehension levels of the nonexpert public must be acknowledged. While the system’s ability to surface latent expert-level themes is a strength, it also risks presenting information in ways that may not be immediately accessible. This highlights a critical future implication: the AI-generated tags should not only drive content recommendation but also inform the simplification and adaptation of health messages. For instance, identifying an expert tag such as “glycemic index management” could trigger the system to prioritize content that explains this concept in simple, actionable terms. This reframes the system as a bridge, not just a filter, ensuring that expert knowledge is translated effectively for diverse audiences and underscoring the continued importance of human oversight in the final presentation of health information, which aligns with emerging research on using LLMs to make complex health knowledge more accessible to the public [23].

### Comparison With Previous Research

This study advances the field of automated health education content annotation in 3 ways. First, in contrast to previous studies that largely relied on narrow datasets or flat label sets, we developed and validated our system against a comprehensive 3-level taxonomy of over 90,000 terms aligned with national public health standards. This represents an ontology that is one order of magnitude larger and deeper than those used in previous LLM tagging studies, such as *International Classification of Diseases (ICD)* coding tasks in Med-PaLM and sentence-level tasks in BioGPT [32,33]

Although other domain-adapted models often rely on full-parameter fine-tuning, which constrains scalability, our use of PEFT via LoRA demonstrates a computationally feasible approach for adapting a 7-billion–parameter model for a highly specific task [20]. This makes the system maintainable and incrementally updatable for local health agencies with limited GPU capacity, which is a critical factor for real-world sustainability that is often highlighted as a barrier to AI adoption in health care.

Most significantly, this study moves beyond offline benchmarks to report the end-to-end deployment of an LLM-assisted tagging service within a governmental health promotion platform. To our knowledge, this is one of the first studies to document the operational feasibility, including subsecond latency and integration via a RESTful API, of such a system in a non-English, multimedia public health context [34].

### Limitations

This study has several limitations. First, the training and test data were sourced from a single municipal corpus, which may limit the generalizability of our findings to other regions or health systems with different content characteristics. Furthermore, the performance on multimedia resources was constrained by the minimal descriptive metadata available, such as videos, audio files, or complex infographics. Given that these formats constitute a growing portion of online health materials, developing multimodal tagging capabilities remains a significant boundary for the current system.

Second, while the expert adjudication was rigorous, it was performed on a random sample (n=50) of “AI Additive” cases; a larger-scale adjudication could further strengthen these findings. The same with our H-H reliability baseline was calculated on a random subset (n=100) of the test data. Although this provided crucial context, a larger sample might offer an even more stable estimate of human performance.

Third, this study focused on the foundational technical validation of the tagging system (ie, its reliability and precision) rather than its downstream usability. As an exploratory check independent of our preliminary evaluation, we conducted a platform poll suggesting high perceived relevance of pushed content among registered users. The results of the questionnaire provide an evidence-based rationale for a subsequent, formal user-experience study. Future research will focus on quantifying this downstream impact using established metrics, which may include the following: task success rate (eg, the percentage of users who successfully find relevant information regarding a specific topic) and time on task (ie, the time taken to find the desired information).

Finally, the broader application of AI in public health warrants careful consideration of potential risks. As with any system trained on large-scale data, biases present in the source corpus could be learned and amplified by the system, potentially leading to the undertagging of content relevant to minority populations or less common health conditions. Although our hybrid pipeline is designed to minimize outright errors, tagging inaccuracies could still occur, necessitating a robust human-in-the-loop validation process, especially for safety-critical information. Furthermore, while our current system processes nonpersonal data, future applications involving user interaction would need to address significant data privacy and governance challenges [35]. These concerns underscore that such AI systems should be viewed as powerful augmentation tools within a framework of continuous human oversight, rather than as fully autonomous agents.

### Conclusions

This study successfully developed and validated an LLM-powered system for automated health content tagging. In response to our objectives, we demonstrated the system’s ability to automate the assignment of a large-scale, 3-tier taxonomy to Chinese HERs. Our evaluation, which moved beyond a simple accuracy assessment, revealed that the AI system functions as a powerful augmentation tool, reliably identifying relevant health topics missed by manual annotators with a high degree of expert-validated accuracy (90% precision). These findings provide a robust technical and methodological framework for implementing AI to enhance, rather than merely automate, precision health communication in public health settings.

## Appendix

Multimedia Appendix 1 Full delphi protocol and process for taxonomy.

Multimedia Appendix 2 Model development design and parameter settings.

Multimedia Appendix 3 Chatgpt coversation.

## Abbreviations

A-H: artificial intelligence–human
AI: artificial intelligence
API: application programming interface
EARS: Early AI-supported Response with Social Listening Platform
GPU: graphics processing unit
HER: health education resource
H-H: human-human
ICD: International Classification of Diseases
IRR: interrater reliability
LLM: large language model
LoRA: low-rank adaptation
NER: named entity recognition
PEFT: parameter-efficient fine-tuning
S-CVI/Ave: scale-level Content Validity Index (averaging method)
SMCHP: Shanghai Municipal Center for Health Promotion
WHO: World Health Organization

## Data Availability

The datasets generated or analyzed during this study are not publicly available due to third-party ownership and data use agreements, but are available from the corresponding author on reasonable request [36].
