# Supplementary material for: Automated Multitier Tagging of Chinese Online Health Education Resources Using a Large Language Model: Development and Validation Study
Source: J Med Internet Res. 2025 Dec 17;27:e83219. doi: 10.2196/83219 (PMC12756663; doi:10.2196/83219)
Supplement: Multimedia Appendix 1 [file jmir_v27i1e83219_app1.docx]

**Full Delphi protocol and process for Taxonomy/Tagging System Design**

**Section S*1.1. Phase 1. Development of the Health Communication Resource Taxonomy***

**Section S1.1.1. Information sources and screening**

We conducted a multisource search to support taxonomy development, including academic literature, Chinese official standards and institutional taxonomy of SMCHP. For academic literature, we designed strategies combining controlled vocabulary and free-text terms related to health education/promotion and core public-health domains. Information on all sources and search strings were listed in Table A1.

**Table S1.** Sources of Information and Their Role in Taxonomy Development

| Database | Type | Exemplar search string | Role in Study |
| --- | --- | --- | --- |
| CNKI, Wanfang | Chinese Databases | 主题=(健康教育 OR 健康促进) AND主题=(分类 OR 本体 OR 主题词 OR 术语) | Sourced literature and standards for screening |
| PubMed, Web of Science | International Databases | (("health promotion"[Title/Abstract] OR "health education"[Title/Abstract]) AND (taxonomy OR ontology OR classification) AND (China OR Chinese)) | Sourced literature and standards for screening |
| Shanghai Municipal Center for Health Promotion (SMCHP) Practice Taxonomy | Institutional Practice Taxonomy |  | Integrated with the evidence-based item pool to ensure operational relevance |
| 22 Official Terminology/Standard Collections | Official Standards |  | Provided an evidence base for taxonomy development |

Inclusion criteria were: (1) national public-health standards/terminologies relevant to health education/promotion; (2) peer-reviewed articles proposing or using public-health taxonomies/controlled vocabularies; (3) Chinese or English; (4) timeframe: **Jan 2013–Mar 2024**. Exclusion criteria were: (1) non-health domains; (2) duplicate or superseded standards; (3) opinion pieces without operational vocabularies.

**Section S1.1.2. Item harvesting and harmonization**

To ensure operational relevance, the evidence-based item pool was integrated with the established three-level practice taxonomy maintained by SMCHP (version 2024-02). Content analysis and semantic normalization were performed to consolidate the variants and align the hierarchical relationships. Rules included:

- Synonym consolidation. For example, merging “HTN”/“hypertension” to the preferred canonical form).
- Polysemy resolution. Assigning surface forms with multiple meanings to the dominant domain and annotating cross-references).
- Parent–child alignment. Moving items to the semantically correct parent category based on definitional fit).
- Terminology mapping. Mapping national standards and commonly used health education vocabularies.

This step produced an initial taxonomy comprising 10 primary (L1), 31 secondary (L2), and 97,233 candidate tertiary (L3) labels prior to expert validation.

**Section S*1.2. Phase 2. Content validation (two-round Delphi)***

**Section S1.2.1. Panel composition and recruitment**

A multidisciplinary panel (n = 20) was convened across public health education, environmental and occupational health, clinical specialties, behavioral science, biostatistics, and informatics/AI. Eligibility required ≥5 years of domain experience and current institutional affiliation. The baseline characteristics and disciplinary mix are summarized in Table A2.

**Table S2.** Baseline characteristics and disciplinary mix of two-round Delphi

| Expert ID | Discipline | Years of experience | Institution type | Education | Round 1 response (Y/N) | Round 2 response (Y/N) |
| --- | --- | --- | --- | --- | --- | --- |
| E01 | Clinical | 16 | Hospital | MD | Y | Y |
| E02 | Clinical | 15 | Hospital | MD | Y | Y |
| E03 | Clinical | 25 | Hospital | MD | Y | Y |
| E04 | Clinical | 11 | Hospital | MD | Y | Y |
| E05 | Clinical | 15 | Hospital | MD | Y | Y |
| E06 | Clinical | 21 | Hospital | MD | Y | Y |
| E07 | Clinical | 27 | Hospital | MD | Y | Y |
| E08 | Clinical | 10 | Hospital | MD | Y | Y |
| E09 | Clinical | 15 | Hospital | MD | Y | Y |
| E10 | Clinical | 16 | Hospital | MD | Y | Y |
| E11 | Informatics-AI | 8 | University | PhD | Y | Y |
| E12 | Informatics-AI | 7 | Industry | PhD | Y | Y |
| E13 | Informatics-AI | 5 | Industry | PhD | Y | Y |
| E14 | Informatics-AI | 9 | Industry | MSc | Y | Y |
| E15 | Behavioral Science | 5 | CDC | PhD | Y | Y |
| E16 | Behavioral Science | 10 | University | PhD | N | Y |
| E17 | Public health | 5 | University | PhD | Y | Y |
| E18 | Public health | 7 | University | PhD | Y | Y |
| E19 | Public health | 16 | University | PhD | Y | Y |
| E20 | Public health | 6 | Governance | MPH | Y | Y |
| Totals/  Response rate |  |  |  |  | 19/20 (96%) | 20/20 (100%) |

**Section S1.2.2. Administration and feedback**

A multidisciplinary panel of 20 experts convened. The panel's expertise covered all key domains relevant to the study, including public health, health education, health communication, food safety, occupational health, health service evaluation, disease prevention, smart health, environmental health, clinical specialties, behavioral science, and large-language-model engineering.

Two Delphi surveys were conducted via email. In each round, the panelists independently rated each item on a 4-point Likert scale for relevance (1 = not relevant; 4 = highly relevant) and provided free-text comments on clarity and hierarchy. The aggregated statistics (median/IQR of ratings and anonymized comments) from Round 1 were fed back to Round 2 to facilitate convergence. Response rates were Round 1 96% for Round 2 100%.

**Section S1.2.3. Metrics and stopping rules (prespecified)**

We prespecified the following process metrics and thresholds:

- Item-level Content Validity Index (I-CVI): proportion of experts assigning scores 3–4 to the item; revision/removal when I-CVI < 0.78.
- Scale-level CVI (S-CVI/Ave): The mean of I-CVIs across items at a given level is interpreted as overall content validity.
- Panel concordance: Kendall’s W with 19 degrees of freedom; stopping rule W ≥ 0.78 with stabilization of item counts across consecutive rounds.
- Significance and uncertainty: We report Kendall’s W, P-values, and 95% confidence bounds computed using Python 3.9.

**Section S1.2.4. Round-wise item flow and finalization**

After Round 1 revisions (revise/merge/delete per I-CVI and comments), the taxonomy was resubmitted for Round 2 confirmation. The process stabilized after two rounds with 10 primary (L1), 34 secondary (L2), and 90,562 tertiary (L3) labels with S-CVI/Ave = 0.91 and Kendall’s W = 0.78, indicating excellent content validity and substantial consensus. Detailed additions/retentions/mergers/deletions by level and round are summarized in **Table A3**.

**Table S3.** Round-wise item flows by level

| Level | Round | Retained | Added | Merged | Removed | Net total |
| --- | --- | --- | --- | --- | --- | --- |
| L1 | Initial (pre-Delphi) |  |  |  |  | 10 |
| L2 | Initial (pre-Delphi) |  |  |  |  | 31 |
| L3 | Initial (pre-Delphi) |  |  |  |  | 97,233 |
| L1 | Round 1 | 8 | 2 | 4 | 0 | 10 |
| L2 | Round 1 | 31 | 8 | 6 | 3 | 36 |
| L3 | Round 1 | 97,233 | 0 | 2,772 | 3,251 | 91,210 |
| L1 | Round 2 (final) | - | - | - | - | 10 |
| L2 | Round 2 (final) | 34 | 0 | 0 | 2 | 34 |
| L3 | Round 2 (final) | 90,562 | 0 | 0 | 648 | 90,562 |

**Section S*A1.3. Final Taxonomy Structure and Validation Metrics***

After two Delphi rounds, the taxonomy stabilized and achieved a high degree of consensus among all panel members. The final validated taxonomy consisted of 10 primary, 34 secondary, and 90,562 tertiary labels. The final validation metrics confirmed the robustness of the taxonomy with an average scale-level Content Validity Index (S-CVI/Ave) of 0.91, indicating excellent content validity.

**Section S*1.4. Statistical definitions***

- I-CVI for item *j*:$I-CVI=\frac{\#expert score 3-4}{\#experts}$
- S-CVI/Ave over a set of *m* items: $S-CVI/Ave=\frac{1}{m}\sum_{j=1}^{m} {I-CVI}_{j}$
- Kendall’s W (coefficient of concordance) was computed on ranks across *k* raters and *n* items; W, 95% CI, and P.
