# Supplementary material for: Automated Multitier Tagging of Chinese Online Health Education Resources Using a Large Language Model: Development and Validation Study
Source: J Med Internet Res. 2025 Dec 17;27:e83219. doi: 10.2196/83219 (PMC12756663; doi:10.2196/83219)
Supplement: Multimedia Appendix 2 [file jmir_v27i1e83219_app2.docx]

**Model Development Design and Parameter Settings**

**Section S2.1 Architectural Design and Rationale**

The automated tagging service was engineered as a three-stage hybrid pipeline (large-language model → named-entity recognition → vector-based standardization), the technical flow diagram is presented in Figure S1.

1. Input Processing: The system ingests textual data from a health resource (article, video, audio, or slide deck), including its title, content, and/or summary description.
2. Generative tagging: The text is processed by a fine-tuned LLM, which first generates a resource summary and then produces an initial broad set of candidate tags based on this summary.
3. Named Entity Recognition (NER) Filtering: The candidate tags are passed to the NER model, which filters out non-core or overly general terms, retaining a refined list of key entities (eg, medical terms).
4. Semantic standardization: The filtered tags are converted to vector embeddings by using a Chroma vector database; and then matched against the pre-indexed vector database via semantic similarity search to collapse to one canonical term from the L3 taxonomy (eg., “fat” and “overweight” to “obese”).
5. Structured Output: The system outputs a final, structured list of standardized tags organized by the L1, L2, and L3 hierarchies.

**Section S2.2 Large Language Model Fine-tuning**

**Section S*2.2.1 Base Model Selection***

The generative component was built on Baichuan2-7B, an open-source Transformer-based LLM. This model was selected because of its state-of-the-art performance on standard Chinese and English benchmarks (eg., C-Eval, MMLU), large training corpus of 2.6 trillion tokens, and open-source license permitting academic research and commercial use. Its proven text-generation and comprehension capabilities have provided a suitable foundation for domain-specific tasks.

**Section S*2.2.2 Parameter-Efficient Fine-Tuning with LoRA***

To adapt the base model to the health communication domain, we employed Low-Rank Adaptation (LoRA), which is a Parameter-efficient Fine-Tuning method. LoRA significantly reduces the computational cost and memory requirements of fine-tuning by freezing the pretrained model weights and injecting a small number of trainable low-rank matrices into the transformer layers. This approach avoids the prohibitive expense of full fine-tuning for a 7-billion-parameter model while maintaining comparable or superior performance. The update to a pretrained weight matrix $W_{0}\in R^{d\times k}$ is represented by a low-rank decomposition:

$$W_{0}+\Delta W=W_{0}+BA,B\in R^{d\times r},A\in R^{r\times k}$$

and rank $r\ll\min\left( d,k \right)$. During the training, $W_{0}$ remains frozen and only matrices *A* and *B* were updated. The forward pass incorporates this update as:

$h=W_{0}x+\Delta Wx=W_{0}x+BAx$,

in which $W_{0}$ and $\Delta W$ held the same constant $x$ (see Figure A1)*.*


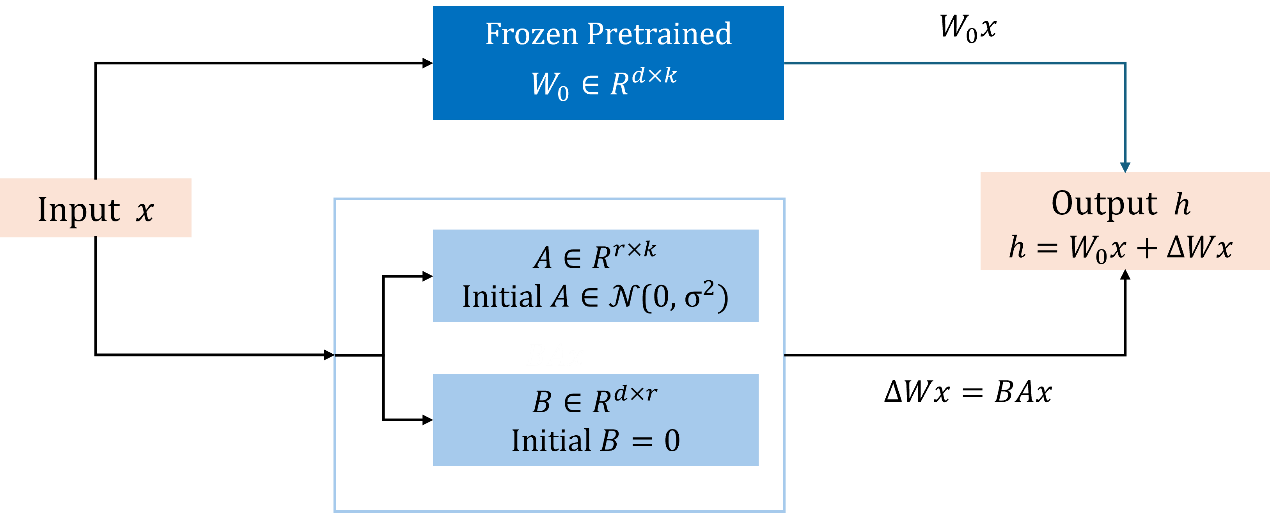


Figure S1. LoRA fine-tuning algorithm principles.

**Matrix** $A$is initialized with a zero-mean Gaussian distribution:

$$A\sim\mathcal{N}(0,\sigma^{2}),$$

**Matrix** $B$is initialized as a **zero matrix**:

$$B=0,$$

ensuring that $\Delta W=0$at the beginning of training and that the pretrained model’s behavior is fully preserved before adaptation begins.

The model was fine-tuned using this method on an expert-annotated dataset containing 10,000 resources.

**Section S*2.2.3 Hyperparameter Configuration***

The model was trained for three epochs, which corresponded to approximately 9,000 optimization steps, and converged in approximately 2 h on a single NVIDIA V100 GPU (80 GB). All pre-trained weights were frozen, except for the LoRA layers. The key hyperparameters see Table S2.

**Table S2.** Key hyperparameters and their values

| Hyperparameters | Values |
| --- | --- |

| LoRA Rank (r) | 16 |
| --- | --- |
| LoRA Alpha (α) | 32 |
| Optimizer | AdamW (β_1_=0.9,β_2_=0.999) |
| Learning Rate | 1 × 10⁻⁴ (with linear decay) |
| Batch Size | 16 |
| Maximum Sequence Length | 1024 tokens |
| Precision | fp16 (mixed precision) |

**Section S2.3 Tag Standardization with Chroma DB**

We implemented the Chroma DB as the vector database for tag standardization. Chroma is an open-source AI-native vector store designed for simplicity and performance, making it well-suited for integration into ML workflows. The entire L3 tag lexicon (N=90,562) was converted into vector embeddings using the *all-MiniLM-L6-v2 model* and indexed to Chroma DB. At the time of inference, candidate tags from the NER filter are vectorized and a k-nearest neighbor search is performed to find the most semantically similar entries in the database. This process ensures that all output tags are mapped to a canonical term within our expert-defined taxonomy, resolving ambiguities and enforcing consistency.

**Section S2.4 Training and Deployment Environment**

The model was trained and deployed on a dedicated server, with the specifications detailed in Table S3.

**Table S3.** Hardware and software specifications for the training and deployment environment.

| Component | Specification |
| --- | --- |

| CPU | 24-Core Processor |
| --- | --- |
| RAM | 256 GB |
| Storage | 2 TB SSD |
| GPU | 1 × NVIDIA V100 (80 GB VRAM) |
| Operating System | Ubuntu 20.04 LTS |
| Key Libraries | PyTorch 2.0, Transformers 4.30, CUDA 11.8 |
