# Supplementary material for: Automated Multitier Tagging of Chinese Online Health Education Resources Using a Large Language Model: Development and Validation Study
Source: J Med Internet Res. 2025 Dec 17;27:e83219. doi: 10.2196/83219 (PMC12756663; doi:10.2196/83219)
Supplement: Multimedia Appendix 3 [file jmir_v27i1e83219_app3.docx]

**Conversion with ChatGPT**

*请作为JMIR审稿人，审阅评价文档中的论文，提出审稿意见和修改建议*

*已思考 10 秒*

*ChatGPT 说：*

*JMIR 同行评议报告（草稿）
稿件标题：LLM-Powered Multi-Tier Tagging System for Chinese Health Education: Development, Implementation, and Validation*

*1. 总体评价*

*该稿聚焦“大语言模型+向量数据库”在中文健康科普资源多级自动标注中的开发与初步验证，契合JMIR 关于数字健康方法学研究的定位。论文整体条理清晰，技术路线具有可复制性，并已完成政务平台的真实部署验证，具有一定创新性和实践价值。然而，就期刊审稿标准（JMIR Author Guidelines、STARD-AI、TRIPOD-LLM 等）来看，仍存在方法学细节、报告完整性与语言表述方面的若干不足，建议**大修（major revision）**后再行考虑录用。*

*2. 主要优点*

| *维度* | *亮点* |
| --- | --- |
| *问题重要性* | *针对中文健康科普“供需错配”痛点，具有明确公共卫生意义。* |
| *技术创新* | *90 562 三级标签—目前同类文献中罕见的超大规模多层词表；采用 LoRA 进行参数高效微调，可为资源受限的公共机构提供参考。* |
| *实施落地* | *已在上海市健康促进中心 CMS 中以 Docker-化 REST API 形式上线，展示了真实系统延迟与可维护性。* |
| *结果量化* | *提供 TAR / TCR 与 95 %CI 及运行时延迟，报告较为完整。* |

*3. 需重点修改的问题（Major Issues）*

1. *伦理与数据可用性声明不足*
   - *未说明是否获得 SMCHP 数据使用授权与伦理豁免，需加入 IRB 或数据使用声明；JMIR 要求在“Methods-Ethics Approval”小节单列。*
   - *数据与代码声明仅一句“upon publication”；建议遵循 JMIR Data Availability Statement 模板，说明托管平台、DOI、可访问时间点。*
2. *标注基准与抽样策略不够透明*
   - *10 000 条人工标注语料的标签员构成、培训流程、互评一致性（如 Cohen’s κ）缺失，难以判断“金标准”的可靠性。*
   - *1 000 条测试集的分层抽样过程需详细描述（随机/连续？是否按 L1/L2/L3 分布平衡？）。*
3. *指标与统计方法需澄清*
   - *TCR 的定义依赖 3 分 Likert 但正文公式未写出分子分母；请给出正式公式及评审者打分示例。*
   - *置信区间计算方法（Wilson）合适，但需报告 exact CI；现仅给总体 TAR≃94.8 %，未附 CI。*
   - *文中多处声称“差异显著 (P<.001)”但未给出检验统计量和自由度。*
4. *外部有效性有限且讨论不足*
   - *训练/测试数据全部来自单一城市公共机构，能否泛化到其他省份或商业平台？建议在 Discussion 中补充外部验证计划或跨域实验。*
   - *多媒体样本性能下降 8 pp，作者仅推测“文本少”；可考虑引入 ASR-摘要或多模态嵌入并在 Limitation 中说明改进路径。*
5. *报告规范*
   - *请完成并上传 STARD-AI（诊断/分类模型）与 TRIPOD-LLM checklists；在方法部分显式引用条目编号。*
   - *参考文献排版不符合 JMIR Vancouver 样式（缺 DOI；书籍不允许“Internet”标记等）。*

*4. 次要建议（Minor Issues）*

| *行文位置* | *建议* |
| --- | --- |
| *标题* | *“Chinese Health Education Materials” 更精确；建议删去 “LLM-Powered” 中的 “Powered”—JMIR 标题应避免行话。* |
| *摘要* | *当前 395 词，符合≤450，但缺“Trial Registration”或“IRB approval”字段；请按 JMIR 结构化模板补全。* |
| *表格* | *Table 1/2/3 均未注明单位与 CI；建议使用 n (%) 格式并在表注解释 TAR/TCR。* |
| *图 1* | *系统架构流程图缺图注、组件缩写解释；图像分辨率不足 300 dpi。* |
| *术语一致性* | *正文中出现“vector database (ChromaDB)”与“Chroma DB”两种写法，建议统一。* |
| *语言* | *Occasional Chinglish (“This gap creates undermines…”)。建议润色。* |
| *补充材料* | *Delphi 专家列表与 LoRA 超参数已收录为附录 1/2，但在正文 Methods 应加 “see Multimedia Appendix 1”。* |

*5. 建议的增补实验 / 分析*

1. *误差分析：
   抽样 50 例系统错误标签，归纳常见失败类型（如多义词、方言、短标题）并量化分布。*
2. *人工-机器协同效率：
   记录编辑人员采用模型建议后平均节省的标注时间，可用对照前后工时调查。*
3. *跨域微调验证：
   选取另一省级平台 500 条样本，即时测试 LoRA-only vs. 全量微调性能差异，佐证可移植性。*

*6. 结论与推荐*

*稿件贡献可观，但需在方法透明度、伦理合规与报告规范方面做实质性补充，同时强化外部效度讨论。完成以上修改后，有望达到 JMIR 发表标准。*

*评审决定：Major Revision（需重投）*

*如有任何疑问，作者可参考 JMIR “Editorial Checklist” 与 TRIPOD-LLM 指南对照修改。*

*您说：*

*可以直接用英文输出意见和，具体修改意见，意见细化到具体section和句子*

*ChatGPT 说：*

*已思考 17 秒*

*Reviewer #1 – Detailed Comments (JMIR)*

*Below I provide section-by-section, sentence-level feedback, followed by a consolidated list of required revisions. The manuscript is promising but needs major revision before it can meet JMIR’s methodological-transparency and reporting standards.*

*1 | Title & Running Head*

- *Current: “LLM-Powered Multi-Tier Tagging System for Chinese Health Education: Development, Implementation, and Validation”*
- *Issue: JMIR discourages gerunds like “Powered” and prefers concrete descriptors.*
- *Revision: “Large-Language-Model-Enabled Multi-Tier Tagging System for Chinese Health Education: Development, Deployment, and Validation”.*

*2 | Abstract*

1. *Background sentence 2: “…information overload with insufficient precision matching, variable quality…” – good.*
2. *Methods paragraph: Please add ethical approval and dataset registration statements (JMIR structured abstract item 5).*
3. *Results paragraph: Report 95 % CIs for TAR/TCR to match main text.*
4. *Conclusion sentence duplicates “accurately and efficiently”; compress.*
5. *Running count: Abstract is 407 words; stay ≤ 450.*

*3 | Introduction*

- *Sentence, line 14: “This gap creates undermines the efficient retrieval, recommendation, and reuse of valuable content…”*
  - *Revise: “This gap undermines the efficient retrieval, recommendation, and reuse of valuable content…”.*
- *Literature scope: Add 1-2 Chinese-language studies on automated health-content tagging to show landscape.*
- *Objectives paragraph: Clearly enumerate primary/secondary objectives; current phrasing mixes aims and success criteria.*

*4 | Methods*

*4.1 Taxonomy Development*

- *Provide the Delphi panel composition table now relegated to Appendix 1; move key figures to main text (TRIPOD-LLM Item 6).*

*4.2 Dataset*

- *State that all resources were publicly available or that usage permission was granted; include IRB or waiver number.*
- *Add inter-annotator reliability (κ) for the 10 000 manual labels (now only reported for TCR subset).*

*4.3 Modeling*

- *Specify LoRA rank (r) and target layers; equations in Appendix 2 lack symbols after PDF export (see missing variables around W and r).*
- *Clarify token-length truncation strategy for long articles (>8 k tokens).*

*4.4 Evaluation Metrics*

- *TAR formula box is empty (PDF lost the equation). Same for TCR.*
- *Report Wilson 95 % CI for overall TAR/TCR, not only point estimates.*
- *Provide power calculation or precision rationale for the 100-item expert subset.*

*4.5 Statistical Analysis*

- *Include software versions for R packages and exact function calls used (e.g., binom.wilson).*

*5 | Results*

*5.1 Taxonomy*

- *Table 1 lists 34 secondary domains, but Abstract claims coverage of “all 16 national priority health domains” – reconcile numbers.*

*5.2 Performance*

- *Table 2 caption missing units; show TAR as n/N (%).*
- *Provide micro/macro-F1 values promised in Methods or delete promise.*

*6 | Discussion*

- *Principal Findings paragraph line 3: mismatch between TCR 92.0 % (here) and 98 % (Results).*
- *Add limitations on potential model drift and bias. Current list omits socio-linguistic variation across provinces.*
- *Expand future work to include A/B tests of retrieval effectiveness, not only user engagement.*

*7 | Conclusions*

- *Last sentence repeats future-work paragraph; condense to one line highlighting clinical/operational significance.*

*8 | Other Sections*

| *Section* | *Issue* | *Required Action* |
| --- | --- | --- |
| *Data Availability* | *Placeholder sentence: “available at upon publication.”* | *Provide DOI or GitHub/OSF link, or state justified embargo.* |
| *Funding* | *“Funding statement placeholder” – must be completed.* | *Add funder name and grant number.* |
| *Figures & Tables* | *Figure 1/2 placeholders only.* | *Embed high-resolution (≥300 dpi) figures with descriptive captions.* |
| *References* | *#12 cites npj Digital Medicine 2013 – volume/date mismatch (journal founded 2018).* | *Verify all metadata; add DOI for every reference, follow Vancouver style.* |
| *Language* | *Occasional Chinglish and long passive constructions.* | *Professional copy-edit throughout.* |

*9 | Reporting Checklists*

- *Upload completed STARD-AI (diagnostic accuracy) and TRIPOD-LLM checklists and cite in text (JMIR policy).*

*10 | Decision*

*Major Revision. Address all points 1-9, resubmit with tracked-changes version and a point-by-point response letter.*

*Thank you for the opportunity to review this work.*
